# Effects of combining robot-assisted therapy with neuromuscular electrical stimulation on motor impairment, motor and daily function, and quality of life in patients with chronic stroke: a double-blinded randomized controlled trial

**DOI:** 10.1186/s12984-015-0088-3

**Published:** 2015-10-31

**Authors:** Ya-yun Lee, Keh-chung Lin, Hsiao-ju Cheng, Ching-yi Wu, Yu-wei Hsieh, Chih-kuang Chen

**Affiliations:** Department of Occupational Therapy and Graduate Institute of Behavioral Sciences, College of Medicine, Chang Gung University, 259 Wenhua 1st Rd, Taoyuan, Taiwan; Healthy Aging Research Center, Chang Gung University, Taoyuan, Taiwan; School of Occupational Therapy, College of Medicine, National Taiwan University, Taipei, Taiwan; Division of Occupational Therapy, Department of Physical Medicine and Rehabilitation, National Taiwan University Hospital, Taipei, Taiwan; Department of Physical Medicine and Rehabilitation, Chang Gung Memorial Hospital at Taoyuan, Taoyuan, Taiwan; School of Medicine, Chang Gung University, Taoyuan, Taiwan

**Keywords:** Stroke, Robot-assisted therapy, Electrical stimulation, Rehabilitation

## Abstract

**Background:**

Robot-assisted therapy (RT) is a widely used intervention approach to enhance motor recovery in patients after stroke, but its effects on functional improvement remained uncertain. Neuromuscular electrical stimulation (NMES) is one potential adjuvant intervention approach to RT that could directly activate the stimulated muscles and improve functional use of the paretic hand.

**Methods:**

This was a randomized, double-blind, sham-controlled study. Thirty-nine individuals with chronic stroke were randomly assigned to the RT combined with NMES (RT + ES) or to RT with sham stimulation (RT + Sham) groups. The participants completed the intervention 90 to 100 minutes/day, 5 days/week for 4 weeks. The outcome measures included the upper extremity Fugl-Meyer Assessment (UE-FMA), modified Ashworth scale (MAS), Wolf Motor Function Test (WMFT), Motor Activity Log (MAL), and Stroke Impact Scale 3.0 (SIS). All outcome measures were assessed before and after intervention, and the UE-FMA, MAL, and SIS were reassessed at 3 months of follow-up.

**Results:**

Compared with the RT + Sham group, the RT + ES group demonstrated greater improvements in wrist flexor MAS score, WMFT quality of movement, and the hand function domain of the SIS. For other outcome measures, both groups improved significantly after the interventions, but no group differences were found.

**Conclusion:**

RT + ES induced significant benefits in reducing wrist flexor spasticity and in hand movement quality in patients with chronic stroke.

**Trial registration:**

ClinicalTrials.gov. NCT01655446

## Introduction

The goal of neurorehabilitation is to restore and maximize physiological function, activities of daily living (ADL), and quality of life for patients with neurological disorders [1]. Robot-assisted therapy (RT) has recently been widely investigated as an effective neurorehabilitation approach that may augment the effects of therapists’ training and facilitate motor recovery [2]. Robotic systems can not only provide external assistance to the paretic limb but also guide accurate, repetitive, and task-specific arm movements [3–5]. RT is cost-effective and labor-saving [6]; moreover, the virtual reality or gaming system that RT is equipped with can increase the motivation level of the participants and enhance engagement in the training programs [7, 8].

Several systematic reviews have been conducted to determine the intervention effects of RT. RT was consistently shown to have significant effects on upper extremity (UE) motor function and strength; however, the evidence of RT effects on ADL and quality of life is limited [5, 9–11]. Although RT has the advantages of providing high-intensity and repetitive arm practice, it cannot directly activate the paretic muscles to enhance motor control of the muscles [4]. This insufficient sensorimotor control of the muscles may thus limit the functional use of the paretic limb in daily living [12].

Neuromuscular electrical stimulation (NMES) has been proposed as one promising adjuvant intervention to RT to directly activate the muscles and improve motor control for patients with stroke [13]. NMES can elicit muscle contraction via electrical stimulation to lower motor neurons [13]. The stimulation current induces depolarization of the peripheral neurons, which subsequently provokes required muscle contraction for target movement. The antidromic (an impulse that travels in the opposite direction to the usual direction) firing of the motor nerve fibers leads to depolarization of the anterior horn cells and results in synaptic remodeling [14]. Furthermore, repetitive and prolonged sensorimotor stimulation coupled with motor training could induce neuroplastic changes in sensorimotor neural network, which may supplement and restore motor and functional abilities of the paretic limb [14]. On the basis of these benefits, NMES could be an encouraging add-on approach to RT for upper limb motor and functional recovery in patients with stroke [12].

Hu and colleagues developed an electromyography (EMG)-driven NMES robotic system to provide assistance to wrist flexion/extension movements in patients with moderately severe stroke. They conducted a series of studies investigating the effects of this device on UE motor recovery for patients with stroke [4, 15, 16]. After 20 sessions of training, participants who received RT with NMES demonstrated significantly greater improvement in UE motor impairment, especially in the proximal part, and in UE motor function than those without NMES [4, 15]. Although RT combined with NMES appeared to reduce spasticity of the wrist and elbow muscles, the beneficial effects were no greater than the RT without NMES group. However, these studies did not further examine whether the improvement in motor impairment could be generalized to functional capacity of daily activities. In addition, the lack of a NMES sham stimulation group might limit the evidence level provided by these results because the participants were not blinded to the group allocation.

In contrast to the unimanual RT employed by Hu and colleagues [4, 15, 16], bimanual RT has been suggested to be a more strategic method to activate muscles of the paretic limb. Bimanual UE training can induce concurrent activation of the homologous cortical regions and facilitate interlimb coordination [17]. Lewis and Perreault (2009) found that initial muscle activation and movement timing of the paretic limb were more synchronized to the nonparetic limb during bimanual RT than during unimanual RT protocol. The synchroneous afferent input from both UEs may thus prime the sensorimotor system and promote subsequent recovery [18]. Because bimanual movements are important and ubiquitous in many daily tasks, bimanual training has been proposed to be an essential component that should be incorportated into upper limb rehabilitation protocols [17].

To extend the findings from previous studies on unimanual RT with NMES, this study employed bimanual RT combined with NMES attempting to augment the therapeutic effects. This study was also designed to blind the treatments to both the evaluators and the participants for reducing potential biases that could be introduced by the knowledge of group assignment. Furthermore, previous studies mainly examined the effects of the hybrid therapy on motor functions, but its influences on ADL and quality of life have not been established. Sivan et al. (2011) recommended that choosing outcome measures from various aspects is important for understanding the full scope of motor training [10]. The purpose of this randomized, double-blind, sham-controlled study was to determine the intervention effects of the hybrid therapy on motor impairment, motor and daily function, and quality of life in patients with chronic stroke. We hypothesized that participants who received bimanual RT with NMES would demonstrate superior performance in the outcome measures compared with those who received bimanual RT with sham NMES stimulation.

## Materials and methods

### Participants

Clinical occupational therapists recruited 39 participants with stroke from five hospitals in Taiwan between 2012 and 2014. The inclusion criteria were (*a*) a first unilateral stroke > 6 months, (*b*) age between 20 and 80 years, (*c*) UE Fugl-Meyer Assessment (UE-FMA) subscore between 25 and 50, (*d*) Mini-Mental State Examination score ≥ 24, and (*e*) did not participate in other research trials during the study period. The exclusion criteria included (*a*) comorbidity with other neurological or psychological disorders, (*b*) severe visuoperceptual impairment (e.g., glaucoma or amblyopia), (*c*) joint arthritis that might prohibit the participant from performing the tasks, (*d*) received botulinum toxin injection within 3 months, and (*e*) in an unstable medical condition.

### Study design

**Fig 1:**
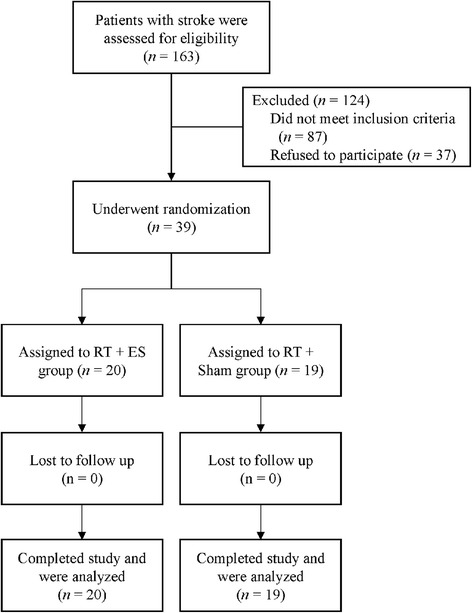
Flow diagram of participants who enrolled and completed the study. Abbreviations: RT, robot-assistive therapy; ES, electrical stimulation

This study was a double-blind, randomized, sham-controlled trial. The Consolidated Standards of Reporting Trials (CONSORT) flow chart is presented in Fig 1. The study procedures were approved by the local hospital and university institutional review boards in Taiwan. After obtaining the written informed consent, the study participants were stratified and randomized by an independent research assistant in our laboratory. The participants were first stratified according to their baseline UE-FMA score (25 to 37 vs 38 to 50) and lesion side, and then were randomly assigned into 1 of the 2 groups: RT with NMES (RT + ES), and RT with sham NMES (RT + Sham). The randomization table for each stratum was generated by an independent research assistant who randomly drew a numbered card representing the group assignment from a concealed envelope. When a new participant was enrolled, the research assistant determined his or her allocated group according to the random table of the stratum. A relevant occupational therapist was then informed about the intervention assigned for the participant.

The participants received their respective interventions for 20 training sessions (90 to 100 minutes/day, 5 days/week for 4 weeks). Besides occupational therapy, the participants were not restricted from participating in other rehabilitation programs, such as physical therapy or speech therapy.

### Intervention

#### RT + ES protocol

The Bi-Manu-Track (BMT; Reha-Stim Co., Berlin, Germany) robotic arm training system was used in this study. The participants sat in front of a height-adjustable table and held the handles of the BMT with the elbow flexed at 90^∘^ and forearms in the neutral position. The robotic training targeted wrist flexion-extension and forearm pronation-supination movements with 3 different training modes: passive-passive (mode 1), active-passive (mode 2), and active-active (mode 3). These 3 modes were chosen in order to progressively improve the movement capacity of the paretic arm. Under the passive-passive mode, both paretic and non-paretic UEs were guided passively by the robotic handle. During the active-passive mode, the non-paretic UE moved the robot handle actively whereas the paretic limb was passively guided by the device. As for the active-active mode, both arms actively move the robot arm against some preset resistance. For each movement, the participants practiced 200 repetitions (5–10 minutes) in mode 1, 750 repetitions (20 minutes) in mode 2, and 50–200 repetitions (5–10 minutes) in mode 3. Movement repetition of mode 3 was depended upon each individual’s capability and was gradually increased throughout the treatment sessions. In each RT treatment session (60–70 minutes), approximately a total of 2000–2300 repetitions were generated for forearm pronation-supination and wrist flexion-extension. After the RT, the participants received an additional 20 to 30 minutes of functional task training to facilitate transferring the acquired movements to daily activities. The selected functional tasks involved forearm pronation-supination or wrist flexion-extension movements, such as twisting a towel or bouncing a ball.

During mode 2 and 3 of RT, NMES (PAS system, model GD 601, OG Giken Co., Okayama, Japan) was also applied to the paretic arm. The stimulation parameters were symmetrical biphasic square waveform with a frequency of 30 pulses per second and a pulse duration of 200 μs. The stimulation intensity was targeted at a muscle contraction level (poor to fair as graded by Manual Muscle Testing Grading System). For the participants who were unable to tolerate the stimulation intensity, the stimulation intensity was adjusted to their maximum tolerance level. Magnetic sensing switches were used to control the on and off time of the stimulator. The sensing switches were placed at the end range of the BMT handle, which was set according to each participant’s movement capability. The magnetic sensing switch would turn on the stimulator when the participants started a movement, and the stimulator was later turned off when the participants reached the end of the movement. The addition of NMES to RT could facilitate the paretic muscles to contract at the appropriate timing. During mode 3 of RT, the participants were instructed to actively contract the muscle along with the NMES in order to work against the resistance. While active muscle contraction would recruit mainly the slow twitch muscle fibers, NMES could activate the fast twitch muscle fibers. Thus, active muscle contraction along with NMES during mode 3 could induce a larger amount of force output to overcome the resistance [19]. For training the wrist flexion-extension movement, the electrodes were placed on the muscle belly of wrist extensors. For the pronation-supination movements, the electrodes were placed over either the forearm supinator or pronator, depending on which muscle is more impaired. Seventy percent of participants had stimulation applied over their supinator muscles, while 30 % of participants received stimulation over the pronator muscle.

#### RT + sham protocol

The intervention protocol for the RT + Sham group was exactly the same as the RT + ES group, except that sham NMES was provided during mode 2 and 3 of RT. For the sham stimulation, the stimulator was turned on but the intensity button was adjusted to 0; thus, there was no current output. The participants were notified that the stimulation intensity was below sensory threshold. Functional task practices were also provided after the RT + Sham training.

### Outcome measures

We selected the outcome measures from various domains, including motor impairment, motor and daily function, and quality of life. The UE-FMA and modified Ashworth scale (MAS) were selected to assess motor impairment. To evaluate motor and daily functions, the Wolf Motor Function Test (WMFT) and the Motor Activity Log (MAL) were chosen. The Stroke Impact Scale 3.0 (SIS) was used to determine the changes in quality of life after intervention.

All the assessments were administrated within 1 week before (pretest) and after the intervention (posttest) by blinded evaluators trained by a senior therapist. The participants were reevaluated 3 months after the posttest to determine whether the treatment benefits were sustained after the intervention. To minimize assessment time, only the UE-FMA, MAL, and SIS were examined at the follow-up test.

#### Motor impairment

The UE-FMA was used to assess the motor impairment after stroke [20]. The UE-FMA includes 21 proximal and 12 distal items, and is scored on a 3-point ordinal scale (0 = cannot perform, 1 = performs partially, 2 = performs fully) with a total score ranging between 0 and 66. The UE-FMA has excellent test-retest reliability, interrater reliability, and validity [20–22].

Muscle spasticity was assessed with the MAS, a method to evaluate spasticity by quickly stretching the targeted muscle [23]. The test-retest reliability, interrater reliability, construct validity, and content validity of the MAS have been reported to be moderate to good for patients with stroke [24–26]. In this study, we examined the MAS of forearm pronators, forearm supinators, wrist flexors, and wrist extensors.

#### Motor and daily function

The WMFT was used to assess the motor function of the paretic UE [27]. The WMFT consists of 17 test items comprising 15 timed and functional tasks and 2 strength-based tasks [28]. It quantifies the total UE movement quality and movement speed with the functional ability scale (WMFT-FAS) and performance time (WMFT-Time), respectively. The participants were instructed to perform each task as quickly as possible, and the time and functional ability for the participant to complete the task was recorded. If a patient spent more than 120 seconds to complete the task, then the score was recorded as 120 seconds. The reliability and validity of WMFT are excellent in patients with stroke [28].

The MAL is a self-reported interview to evaluate how often (amount of use [AOU]) and how well (quality of movement [QOM]) an individual with stroke uses the paretic hand in daily life [29]. It contains 30 questions, and each question is scored from 0 to 5, with a higher score indicating a greater amount of use or better quality of arm movement. The test-retest reliability and criterion validity of MAL are excellent in patients with stroke [30, 31]

#### Quality of life

The SIS is a self-report questionnaire that assesses quality of life in patients with stroke [32]. The SIS consists of 59 items scored on a 5-point Likert scale with a higher score indicating better quality of life. The SIS contains 8 domains: strength, memory, emotion, communication, basic and instrumental ADLs, mobility, hand function, and participation. For this study, we focused on the domains that are specifically related to physical function, that is, strength, ADLs, mobility, and hand function. The reliability and validity of SIS is moderate to excellent in patients with chronic stroke [33, 34].

### Data analysis

To compare the baseline characteristics between the 2 groups, the χ^2^ test and independent sample *t* test were used to analyze categorical and continuous variables, respectively. Group (RT + ES vs. RT + Sham) × time (pretest vs. posttest [vs. follow-up]) repeated measures analysis of variance (ANOVA) were used to examine the intervention effects. The Bonferroni post hoc test was performed if an interaction or a main effect was found. The significance level was set at .05. We also calculated the effect size using partial eta-squared (η^2^) for each outcome variable. A larger effect size represented a greater magnitude of treatment effect. An effect were considered large at η^2^ value ≥ .138, moderate at η^2^ ≥ .059, and small at η^2^ ≥ .010 [35]. Statistical analyses were performed with SPSS 19.0 (SPSS Inc, Chicago, Illinois).

## Results

The baseline characteristics of the participants are summarized in Table 1. There were no statistically significant differences between the 2 groups. Group differences of all the outcome measures at baseline were also analyzed. The mobility domain of the SIS revealed a significant group difference (*p* = .039) at baseline, while no significant differences were found in other outcome measures (Table 2).

**Table 1:** Baseline characteristics of the participants

	RT + ES (*n* = 20)	RT + Sham (n = 19)	*P* Values
Sex (male/female)	15/5	14/5	.925
Age (years)	54.07 (11.85)	53.75 (9.11)	.924
Months since stroke	25.40 (17.09)	27.95 (16.20)	.636
Side of brain lesion (left/right)	9/11	10/9	.634
Type of stroke (hemorrhage/ischemic)	10/10	8/11	.621
Handedness (left/right)	0/20	0/19	–
UE-FMA	30.70 (9.76)	26.63 (10.58)	.219
MMSE	27.10 (1.83)	27.47 (1.78)	.522
Time of PT (hours/week)	3.56 (3.45)	2.45 (3.26)	.310

Data are presented as mean (standard deviation)

*RT* robot-assistive therapy, *ES* electrical stimulation, *UE*-*FMA* Fugl-Meyer Assessment upper extremity subscore, *MMSE* Mini-Mental State Examination

**Table 2:** Descriptive and inferential statistics of the clinical outcome measures (with follow-up)

Outcomes	RT + ES (*n* = 20)			RT + Sham (*n* = 19)			Repeated measure ANOVA		
	Pretest	Posttest	Follow-up	Pretest	Posttest	Follow-up	*F*	*P*	Partial η^2^
*Motor impairment*									
UE-FMA	30.70 (9.76)	34.60 (9.79)**	32.90 (8.75)	26.89 (10.66)	30.68 (10.02)**	29.21 (9.25)**	.010	.955	<.01
MAS									
Forearm pronator	1.10 (0.58)	1.18 (0.63)	--	1.37 (0.70)	1.29 (0.75)	--	.974	.330	.026
Forearm supinator	0.05 (0.22)	0.05 (0.22)	--	0.05 (0.23)	0.13 (0.40)	--	.366	.549	.010
Wrist flexor	1.35 (0.59)	1.08 (0.49)**	--	1.03 (0.66)	1.13 (0.64)	--	4.161	.049^*^	.101
Wrist extensor	0.28 (0.50)	0.18 (0.44)	--	0.21 (0.42)	0.21 (0.42)	--	.949	.336	.025
*Daily function*									
WMFT									
Time	7.39 (3.26)	6.72 (3.18)	--	8.50 (4.06)	7.30 (2.50)	--	.545	.465	.015
FAS	2.54 (0.52)	2.76 (0.55)**	--	2.35 (0.61)	2.43 (0.61)**	--	5.971	.019^*^	.139
MAL									
AOU	0.57 (0.57)	0.92 (0.81)**	0.71 (0.65)	0.54 (0.70)	0.82 (1.01)**	0.69 (0.82)	.165	.848	.004
QOM	0.47 (0.53)	0.86 (0.75)**	0.61 (0.56)	0.51 (0.63)	0.79 (0.95)**	0.59 (0.69)	.351	.705	.009
*Quality of life*									
SIS Physical Function	58.87 (9.57)	64.43 (12.34)**	57.43 (12.54)	56.57 (11.33)	64.19 (14.12)**	54.17 (8.40)***	.401	.631	.012
Strength	38.75 (8.98)	44.69 (16.26)	41.56 (14.38)	37.17 (17.36)	44.74 (16.96)	40.46 (15.92)	.065	.937	.002
ADL	77.94 (17.68)	81.76 (15.86)	79.96 (15.99)	75.96 (14.56)	76.37 (15.32)	77.16 (13.00)	.537	.587	.014
Mobility	93.21 (5.86)****	93.61 (6.50)	93.20 (5.67)	82.69 (19.98)	89.23 (15.07)	90.88 (14.36)**	4.372	.016*	.106
Hand function	26.78 (22.07)	38.42 (29.29)	50.32 (34.31)	24.93 (21.14)	42.62 (26.43)**	25.02 (20.79)***	4.992	.014^*^	.128

Data are presented as mean (standard deviation)

*RT* robot-assistive therapy, *ES* electrical stimulation, *UE*-*FMA* Fugl-Meyer Assessment upper extremity subscore, *MAS* modified Ashworth scale, *MAL* Motor Activity Log, *AOU* amount of use, *QOM* quality of movement, *WMFT* the Wolf Motor Function Test, *FAS* functional ability scale, *SIS* Stroke Impact Scale 3.0, *ADL* activities of daily living

* Significant group × time interaction revealed by repeated measures ANOVA (*p* < .05)

** Significantly different from pretest (*p* < .05)

*** Significantly different from the posttest (*p* < .05)

**** Significantly different from the RT + Sham group (*p* < .05)

### Motor impairment

For the UE-FMA score, repeated measures ANOVA showed no significant group × time interaction (*F*_2, 74_ = .010, *p* = .955, η^2^ < .001). A significant time main effect was found (*F*_2, 74_ = 11.852, *p* = .001, η^2^ = .243), with no significant group main effect (*F*_1, 37_ = 1.635, *p* = .209, η^2^ = .042). This result showed that the UE-FMA score improved significantly after the bimanual RT intervention, but the 2 groups did not differ in the amount of improvement (Table 2).

For the MAS score of the wrist flexor, there was a significant group × time interaction (*F*_1, 37_ = 4.161, *p* = .049, η^2^ = .101), with no significant time or group main effect (Table 2). Post hoc analyses of the interaction revealed that while the MAS of wrist flexor decreased significantly for the RT + ES group (*p* = .017), no significant change was found for the RT + Sham group (*p* = .508). Asides from the wrist flexor, no significant changes were observed for the MAS scores of forearm pronators, forearm supinators, and wrist extensors after the interventions.

### Motor and daily function

The RT + ES and RT + Sham groups improved significantly in the WMFT-Time after the interventions (*F*_1, 37_ = 6.874, *p* = .013, η^2^ = .157), but no significant group × time interaction or group main effect was found. Both groups demonstrated comparable improvements in the WMFT-Time performance. A different pattern of improvement was observed for the WMFT-FAS score. The results of the WMFT-FAS revealed a significant group × time interaction (*F*_1, 37_ = 5.971, *p* = .019, η^2^ = .139) and a significant time main effect (*F*_1, 37_ = 28.910, *p* < .001, η^2^ = .439), but no group main effect (*p* = .166). Although both groups improved significantly after treatment, the RT + ES group demonstrated greater improvement than the RT + Sham group (Table 2). Additional analysis for the hybrid effects on each WMFT item revealed that compared with the RT + Sham group, the RT + ES group had significantly greater improvements in the *Lift can* and *Lift pencil* tasks.

Repeated measures ANOVA showed no significant group × time interaction on the MAL AOU and QOM subscales. Both groups demonstrated significant within-group improvement after intervention on the AOU (*p* < .001) and QOM (*p* < .001), but these improvements did not persist to the follow-up examination. There were also no significant differences in the amount of improvement between the 2 groups.

### Quality of life

For the SIS physical function score, no significant group × time interaction or group main effect was found. A significant time main effect (*p* < .001) was observed, showing that the SIS physical function first improved after intervention but declined at the follow-up examination.

Analysis of the SIS strength and ADL domains revealed no significant group × time interaction, with no significant group or time main effect. Interestingly, we observed a significant group × time interaction (*p* = .017) and a significant time main effect (*p* = .012) for the mobility domain of SIS. Post hoc analysis revealed that although the mobility of the RT + ES group did not change significantly among the 3 test sessions, the mobility level of the RT + Sham group improved after the intervention and continued to improve at the follow-up test. For the hand function domain, a significant group × time interaction (*p* = .014) was also observed, with a significant time main effect (*p* = .014) but no group main effect (*p* = .261). The RT + ES group improved after the intervention and continued to improve at the follow-up test (close to significant, *p* = .056); the RT + Sham group, on the other hand, appeared to improve immediately after the intervention, but then the performance declined at the follow-up examination (Table 2).

## Discussion

This is the first randomized, double-blind, sham-controlled study that examined the intervention effects of bimanual RT combined with NMES on motor impairment, motor and daily functions, and quality of life. The evidence level of the present study was enhanced by including a sham group and blinding the evaluators and participants to the group allocation. The RT + ES group was evident to have more beneficial effects on muscle spasticity of the wrist flexors and the motor function in the aspect of movement quality (i.e., the WMFT-FAS) than the RT + Sham group. Both groups improved significantly after training in the motor impairment (i.e., UE-FMA) and daily function (i.e., MAL), but there were no significant group differences. The RT + ES group also demonstrated significant benefits in the hand function domain of the quality of life as measured by the SIS.

One main finding of the study was that applying NMES in addition to RT could significantly reduce muscle spasticity of the wrist flexors. A systematic review found that the effects of RT on muscle spasticity were inconsistent [11], suggesting that the reduction in spasticity observed in this study was probably attributed to the adjuvant treatment effects of NMES. Since spasticity is associated with anomalous muscular properties and/or supraspinal alterations, NMES may decrease muscle over-activation via the mechanism of reciprocal inhibition and induce plasticity to the spinal cord pathways [14]. Peripheral stimulation from the NMES activates Ia afferents, which enter the spinal cord through the dorsal horn. One branch of the Ia afferent synapses with the Ia inhibitory interneurons that innervate the alpha motor neurons of the antagonist muscle. Because the interneurons are inhibitory, they prevent firing of the alpha motor neurons and thus reduce the over-activation of the antagonist muscle and decrease spasticity [36–38].

In the current study, the active electrodes were placed over the wrist extensor muscles during RT; therefore, a reduced spasticity was observed for the wrist flexors. Inconsistent with our findings, Hu et al. (2014) did not observe that RT + ES training induced specific benefits for wrist and elbow MAS scores compared with RT alone [4]. The discrepancy could be related to differences in the type of robot training, participant characteristics, amount of therapy (number of movement repetitions), and stimulation modes of NMES. Hu and colleagues provided unilateral RT, while bimanual RT was employed in the current study. The participants in Hu’s study were in general younger but had longer stroke onset in comparison to the participants of the current study. Furthermore, the amount of arm practice differed significantly between the 2 studies. The participants in Hu’s study practiced around 70 repetitions of wrist extension/flexion (14 trials of movement with 5 cycles of wrist movement in each trial) in each training session, while around 1000 repetitions of wrist flexion/extension were given to the participants in this study. As for the stimulation mode of NMES, Hu et al. (2014) used an EMG-triggered NMES device in conjunction with RT, and we used a cyclic stimulation approach to elicit muscle contraction. It is possible that cyclic stimulation can stimulate the spinal interneurons more consistently and enhance synaptic remodeling to reduce muscle spasticity [14]. Future studies may consider investigating the differential treatment effects of the above factors.

Although a significant change in the spasticity of the wrist flexor was observed, no obvious changes were observed for the forearm pronator and supinator of the UE. One factor may be associated with the specificity of the stimulated muscle. The selection of supination/pronation stimulation depended on the primary movement limitation of the participants; thus, not everyone had stimulation over the same muscles. Furthermore, those muscles are deeper in the forearm. Therefore, the stimulation of supination and pronation may not be specific enough to induce changes in muscle spasticity.

As for the measures of motor function, the RT + ES and RT + Sham groups both showed an improvement in WMFT-Time and WMFT-FAS after the interventions. Although the 2 groups did not differ in the amount of improvement in WMFT-Time, the RT + ES group demonstrated significantly greater improvement in the WMFT-FAS than the RT + Sham group. The benefit on WMFT-FAS was probably attributed to an augmented intervention effect of bimanual RT and NMES. It is possible that through repetitive muscle contraction and the sensory inputs from RT and NMES, the brain is “taught” how to contract appropriate muscles to produce smooth movements [12, 14]. Via intensive and repetitive bimanual practice, the participants learned to shape the movement trajectory of the paretic arm as symmetrically as the non-paretic limb. With the help of NMES, the participants learned to activate the paretic muscles at an appropriate timing and produce a proper amount of force. The most salient performance might be demonstrated by lifting the can and pencil tasks as evaluated by WMFT. Therefore, the combination of these 2 intervention approaches improved the overall movement quality performance, as measured by the WMFT-FAS.

Owing to the non-significant between-group differences, the observed improvement in WMFT-Time was probably attributed to the addictive effects of RT and functional task practice but not specifically related to NMES. Only a few studies have investigated the effects of RT + ES on WMFT-Time, and they all showed that RT + ES significantly reduced the time to perform the tasks [12, 15]. However, these studies did not have a dose-matched comparison group to demonstrate that the observed improvement was specifically the result of an augmented effect of RT + ES. Our study suggested that the hybrid therapy did not induce specific benefit on WMFT-Time when compared with the RT + Sham group. Studies investigating the effects of RT showed indefinite results on the WMFT. Most case-controlled studies of RT showed a significant reduction in WMFT-Time, whereas randomized controlled trials consisting of 2 or more groups revealed no significant differences in WMFT performance between RT training and dose-matched conventional therapy [39–41]. In addition, the improvement of WMFT-Time performance after pairing peripheral sensory stimulation with task-specific training was not significantly greater than the group receiving task-specific training only [42]. These observations, together with the findings of the present study, suggest that NMES combined with RT or task-specific training, and task-specific training alone might positively affect hand motor function for patients after stroke. Hence, RT, a task-oriented approach, and combined therapy may all be plausible options for clinicians to improve movement speed of the paretic limb after stroke.

The results revealed that both groups improved significantly on UE-FMA and MAL without significant group differences. One reason for the lack of group differences could be related to the amount of movement practiced with the BMT. Treatment dosage has been recognized as one of the key elements for motor improvement after an intervention [43, 44], and RT is a treatment method that can provide intensive training to the paretic hand. Therefore, because the RT + ES and RT + Sham groups in the current study received an RT-based intervention with the same amount of arm and hand practice, the treatment-induced improvement in UE-FMA did not differ between the groups. Inconsistent with our findings, Hu et al. (2014) found that the EMG-triggered NMES RT group had significantly better performance in the UE-FMA than the RT group [4]. The differential findings between studies could be related to various factors, such as distinct patient characteristics, implementation of RT, or application of NMES. It is also possible that EMG-driven NMES took into account the capability of the participants during training and thus induced superior treatment effects on UE-FMA.

For the assessment of physical function in SIS, very interesting findings were observed in the hand function and mobility domains. Regarding the changes in the hand function domain, the participants in the RT + ES and RT + Sham groups reported less difficulty using their paretic hand to perform functional tasks at posttest. More importantly, the participants in the RT + ES group showed even less difficulty at the follow-up examination. Although we did not have follow-up data for the MAS and WMFT-FAS, it is possible that the reduction in muscle spasticity of wrist flexor and the successfulness in using the paretic hand enhanced self-efficacy (e.g., while lifting objects) and further motivated the participants to continue using the paretic hand after the intervention [45, 46]. The persistent use of the paretic limb may thus reduce the perceived difficulty in using the paretic hand to complete functional tasks. Conversely, the hand function of the participants in the RT + Sham group decreased back to baseline performance at the follow-up examination, suggesting that these participants might have discontinued using their paretic arm after the intervention ceased. It is possible that the participants in the RT + Sham group could not use the paretic arm to perform motor tasks effectively and efficiently, which discouraged them to frequently use the paretic arm after intervention.

Besides from the hand function domain, a group × time interaction was observed for the mobility domain in SIS. Although the mobility in the RT + Sham group improved at the posttest and the follow-up test, the RT + ES group did not change significantly over time. The improvement in home and community mobility after RT + Sham intervention could be associated with multiple factors, such as the improved interlimb coordination [47, 48] or the additional physical therapy that the participants received. Distinct from the participants in the RT + Sham group, the participants in the RT + ES group already had a high mobility level at baseline, which might have hindered the potential training benefits on mobility (ceiling effect).

One limitation of the study is that not all the outcome measures were assessed at follow-up. When designing the study, we purposefully reduced the number of outcome measures for the follow-up evaluation in order to minimize the assessment time. Nevertheless, having follow-up data for all the outcomes might further help us interpret the findings of the hybrid therapy. Another potential confounding factor of the study is the stimulation intensity of NMES. In the study, stimulation intensity was set at the poor to fair level of the Manual Muscle Testing Grading System (according to the tolerance level of the participant). The findings indicate that this level of stimulation intensity could significantly reduce wrist flexor muscle spasticity and improve movement quality of the paretic hand. Future research could enhance the stimulation intensity, which might further elicit muscle contraction and lead to greater improvement in hand motor impairment and ADLs. Future studies could also adjust the stimulation frequency, pulse duration, and waveform to maximize the stimulation effects of NMES. Furthermore, studies that compare the intervention effects of RT + NMES, RT only, and NMES only will provide more insight to the benefits of the hybrid therapy. The last confounding factor of the study is that the amount of therapy or exercise outside the intervention period was not controllable. We recorded the time for physical therapy and found no significant group differences (Table 1). Nevertheless, extra therapy or exercise that participants engaged in might bias the results of the study.

## Conclusion

This randomized, double-blind, sham-controlled study demonstrated that combining peripheral NMES with bimanual RT is a promising intervention method to reduce wrist flexor muscle spasticity and improve hand movement quality. Compared with RT + Sham stimulation, the RT + ES hybrid approach did not induce additional benefits on the UE-FMA, MAL, and the SIS strength and ADL domains. Future studies can adjust the stimulation modes and determine the dosage effects of the RT + ES therapy.

## Abbreviations

ADL: activities of daily living
AOU: amount of use
EMG: electromyography
FAS: functional ability scale
FMA: Fugl-Meyer Assessment
MAL: Motor Activity Log
MAS: modified Ashworth scale
NMES: neuromuscular electrical stimulation
QOM: quality of movement
RT: robot-assisted therapy
SIS: Stroke Impact Scale 3.0
UE: upper extremity
WMFT: Wolf Motor Function Test
